# Twenty‐year medication use trends in first‐episode bipolar disorder

**DOI:** 10.1111/acps.13504

**Published:** 2022-10-12

**Authors:** Juulia Poranen, Aura Koistinaho, Antti Tanskanen, Jari Tiihonen, Heidi Taipale, Markku Lähteenvuo

**Affiliations:** ^1^ Department of Forensic Psychiatry University of Eastern Finland, Niuvanniemi Hospital Kuopio Finland; ^2^ Department of Clinical Neuroscience Karolinska Institutet Stockholm Sweden; ^3^ Center for Psychiatry Research Stockholm City Council Stockholm Sweden; ^4^ School of Pharmacy University of Eastern Finland Kuopio Finland

**Keywords:** antipsychotics, bipolar disorder, medication trends, mood stablizers, pharmacoepidemiology

## Abstract

**Objectives:**

To study the medication use patterns in patients newly diagnosed with bipolar disorder (BD) in Finland during the past 20 years.

**Methods:**

All persons diagnosed with BD between 1996 and 2018, aged 16–65 years, with no previous BD diagnosis were identified from nationwide Finnish registers (*N* = 26,395). The point prevalences of medication use were observed up until 5 years after the first diagnosis. Five sub‐cohorts according to calendar year of first diagnosis were also formed and the prevalence of medication use was compared between sub‐cohorts 3 months after diagnosis. Medication data were modeled with the PRE2DUP‐method using dispensing data.

**Results:**

The prevalence of overall medication use declined during the 5‐year follow‐up period in the total cohort. The highest prevalence of use was seen 3 months after diagnosis for the three main medication classes–antidepressants (40.8%), antipsychotics (30.8%) and mood stabilizers (29.2%). The prevalence of lithium use varied between 5.9% and 6.5% during the 5 years in the total cohort, and the lowest prevalence of use at 3 months was seen in sub‐cohort diagnosed in 2016–2018 (4.1%) versus 12.1% in 1996–2000 sub‐cohort. The prevalence of benzodiazepine use was between 12.4% and 13.5% and the prevalence of Z‐drugs was between 7.3% and 7.9% during the 5 years. The prevalence of long‐acting injectable antipsychotic (LAI) use was the highest in patients diagnosed in 2016–2018, although still only 0.8%.

**Conclusions:**

(i) The use of antidepressants is too prevalent, (ii) the use of lithium is declining and needs to be increased, and (iii) LAIs are markedly underutilized as compared to their oral counterparts.

**Significant outcomes**

- The prevalence of antidepressant use was high during the five‐year observation period following the first diagnosis of bipolar disorder.
- The use of lithium significantly declined over the past two decades and lithium was also widely underused at least in the first 5 years following diagnosis.
- Long‐acting injectable antipsychotics were markedly underutilized as compared to their oral counterparts.

**Limitations**

- All diagnoses are based on data collected from digital registers and no patients were met or diagnosed by the study team, which therefore leads to a slight chance of misdiagnoses and selection bias of the study population that the study team was not able to control for.
- There is an unavoidable possibility that some medications dispensed have not been used by the patients.

## 1 INTRODUCTION

Bipolar disorder (BD) is a serious, chronic, and often reoccurring psychiatric disorder affecting approximately 1% of the world's population and as much as 2%–3% when considering the broader spectrum of bipolar disorders.^1^, ^2^ BD is typically characterized by alternating manic and depressive episodes as well as episodes of mixed states (in which the symptoms of mania and depression occur simultaneously) and it is often associated with severe clinical, psychosocial and cognitive decline as well as increased mortality with suicide being one of the main causes of death.^1^, ^2^, ^3^ The treatment of BD is often complex because of its wide range of clinical manifestations and their tendency to change significantly over time.^4^ The treatment must include the management of acute phases as well as maintenance treatment and prophylaxis of relapses, which often leads to many drug trials and polytherapy to achieve sufficient therapeutic response.^1^, ^4^

Pharmacological treatment is the cornerstone of managing the acute phases in BD. Currently, Clinical Practice Guidelines (CPGs) such as CANMAT, ISBD and NICE support the use of second‐generation antipsychotics (SGAs) such as aripiprazole, quetiapine, olanzapine and risperidone for the treatment of acute bipolar mania as well as lithium and valproate for the long‐term treatment of BD.^2^, ^4^, ^5^, ^6^, ^7^

While the treatment recommendations for bipolar depression are more controversial and less studied,^3^, ^8^ most CPGs agree on recommending against the use of antidepressants as a monotherapy and some even as an adjunctive.^8^, ^9^, ^10^ Importantly, despite this discouragement towards antidepressant use, studies show that antidepressants are prescribed commonly for patients with bipolar depression.^11^, ^12^, ^13^ Instead of antidepressants, recent CPGs recommend quetiapine, lurasidone, lithium, and lamotrigine as first‐line treatment options for the treatment of bipolar depression.^6^, ^7^ For maintenance therapy of BD, the recommendations in CPGs as well as in several research studies are more consistent–most view lithium as the superior treatment choice either as monotherapy or adjunctive.^2^, ^3^, ^14^, ^15^, ^16^ Despite this, studies have found that the prescribing of lithium has decreased significantly in BD patients over time.^12^, ^13^ In addition to sufficient pharmacotherapy, it is also recommended to offer psychotherapy and psychoeducation for patients with BD to reduce relapse and reoccurrence of acute episodes.^17^

Despite the major advances in clinical knowledge about the efficacy and safety of medications used for managing BD, there are still numerous challenges to overcome, one of which seems to be the divergence between evidence‐based treatment recommendations and the real‐world adherence to the evidence. While several studies have investigated the trends in pharmacological treatment of BD in different settings, many have relied on prescription and not usage data (covering also pharmacy dispensings and avoiding the issue with primary non‐adherence, that is, not purchasing the prescribed drug)^12^, ^13^, ^18^ or been limited in their inclusiveness and observation periods. A previous Danish study provided a comprehensive overview on the prescription patterns in first‐episode bipolar disorder, using dispensing data.^19^ To provide more recent data on usage trends, this study aimed to investigate the trends and changes in the real‐world usage of medication in newly diagnosed patients with bipolar disorder during the period of 1996 to 2018 using nationwide data from Finnish registers.

## 2 METHODS

### 2.1 Study population

The base cohort included all persons diagnosed with bipolar disorder (ICD [International Classification of Diseases]‐10 F30–F31) between the years 1996–2018 and between the age of 16–65 years, with no previous BD diagnosis recorded in Finland (*n* = 51,621). These persons were identified from nationwide Finnish registers: inpatient care (since 1996) and specialized outpatient care (since 1998, The Care Register for Health Care, maintained by National Institute of Health and Welfare), sickness absence (since 2004, maintained by Social Insurance Institution, SII) and disability pensions (since 1996, identified from registers of SII and Finnish Centre for Pensions). The specialized outpatient care refers to public outpatient clinics at hospitals. Private psychiatrists are not included. Sick leaves and disability pension registers include patients from primary care and those treated by private psychiatrists.

The Care Register for Health Care includes all visits to hospitals and specialized outpatient care. Sickness absences included sick leaves of 14 days or more. Diagnoses from primary care were only obtainable through sick leaves and disability pensions. The Prescription Register data includes all reimbursed medication dispensings from Finnish pharmacies to all residents since 1995. Prescription Register data includes the date of dispensing, ATC code, product information (name of drug product, strength, package size), the number of packages dispensed and dispensed amount in Defined Daily Doses (DDDs) as defined by the WHO.

From the base cohort, persons with a previous BD diagnosis and those who had used any antipsychotic drug (ATC [Anatomical Therapeutic Chemical]‐code N05A, excluding lithium N05AN01) or any mood stabilizer (N03AF01 carbamazepine, N03AG01 valproate, N03AX09 lamotrigine and N05AN01 lithium) during the previous year before the diagnosis were excluded. Also, persons with diagnosis of schizophrenia (F20), schizoaffective disorder (F25) or dementia (F00‐F03, G30) before bipolar disorder diagnosis were excluded. After these exclusions (*N* = 25,226), *N* = 26,395 persons meeting the criteria were included in this study.

Each person in Finland has a unique personal identification number that enables the reliable tracing of all individuals from various registers over time.

### 2.2 Exposure

Drug use information in the Prescription register data is categorized according to the ATC classification. Antidepressants were defined as ATC‐code N06A; antipsychotics were defined as ATC‐code N05A excluding lithium (N05AN01) and mood stabilizers were defined as any of these four: carbamazepine (N03AF01), valproate (N03AG01), lamotrigine (N03AX09), lithium (N05AN01). Mood stabilizers were further divided into lithium (N05AN01) and anticonvulsants including carbamazepine (N03AF01), valproate (N03AG01), lamotrigine (N03AX09). In addition, antidepressants were also divided into subcategories: SSRIs (selective serotonin reuptake inhibitors) were defined as ATC‐code N06AB, SNRIs (serotonin‐norepinephrine reuptake inhibitors) were defined as venlafaxine (N06AX16), milnacipran (N06AX17) and duloxetine (N06AX21), mirtazapine was defined as ATC‐code N06AX11, and the group ‘other antidepressants’ included the rest of medications belonging to N06A. Most common specific antipsychotics were also identified (oral if not otherwise stated): quetiapine (N05AH04), olanzapine (N05AH03), aripiprazole (N05AX12), risperidone (N05AX08), levomepromazine (N05AA02) and then the rest were grouped as ‘other antipsychotics’. LAIs were separately categorized based on drug form. Benzodiazepines were defined as ATC‐codes N05BA and N05CD and Z‐drugs as ATC‐code N05CF.

Drug use periods, that is, when drug use started and ended were modeled with PRE2DUP method.^20^ The modeling method is based on dispensing dates, amounts dispensed and drug‐specific parameters, which define clinically relevant upper and lower limits for daily dose. The method also takes into account stockpiling, days spent in hospital care (when drug use is not recorded in the Prescription Register as drugs are provided by the caring unit) and personal regularity of use.

### 2.3 Statistical analysis

We studied the point prevalence of medications used during a five‐year period after the first recorded diagnosis. During the five‐year follow‐up period, the use of medication was observed for every individual included in the study population (i) every 3 months during the first year (3, 6, 9, and 12 months after diagnosis) and (ii) every 6 months after the first year and up until 5 years after diagnosis, leading to a total of 13 time points.

At each time point, the prevalence of use was assessed as two‐week point prevalence of use. As drugs provided during hospital care are not recorded in the Prescription Register, only those persons who were alive, not censored due to end of data linkage or diagnosis of schizophrenia spectrum disorder (F20–F29), and who were not hospitalized for more than 5 days of a specific 14‐days evaluation period were included at that time point.

This exclusion/ censoring leads to the study population being slightly different at each time point. The study population was also divided into sub‐cohorts depending on when they had received their first recorded diagnosis of bipolar disorder, namely 1996–2000 (sub‐cohort 1), 2001–2005 (sub‐cohort 2), 2006–2010 (sub‐cohort 3), 2011–2015 (sub‐cohort 4) and 2016–2018 (sub‐cohort 5).

Prevalence of use at 3 months after the diagnosis was compared between the sub‐cohorts to observe time trends in early treatment patterns. Prevalence of use between different time points and between the sub‐cohorts were compared with 95% confidence intervals.

The cumulative incidence of medications used during the first year after diagnosis was also studied stratified by the calendar year of the first diagnosis.

The statistical analyses were performed using IBM SPSS Statistics for Windows version 27.0 (IBM Corp, USA) and data management with SAS version 9.4 (SAS Institute Inc, USA).

### 2.4 Ethics

The research project was approved by pertinent institutional authorities at the Finnish National Institute for Health and Welfare (permission 635/5.05.00/2019), the Social Insurance Institution of Finland (31/522/2019), Finnish Centre for Pensions (19023) and Statistics Finland (TK‐53‐569‐19).

## 3 RESULTS

### 3.1 Study population and sub‐cohorts

Of the study population (*N* = 26,395), 54.9% (*N* = 14,483) were women and 45.1% (*N* = 11,912) men. Mean age at the time of first recorded diagnosis of bipolar disorder for the cohort was 38.2 years (SD 13.0). The study population included patients diagnosed between the years 1996 and 2018, and they were divided into sub‐cohorts depending on the year of initial recorded diagnosis. The characteristics of the study population and sub‐cohorts are shown in Table 1.

**TABLE 1:** Baseline characteristics of the study cohorts

	Total cohort	Sub‐cohort 1 1996–2000	Sub‐cohort 2 2001–2005	Sub‐cohort 3 2006–2010	Sub‐cohort 4 2011–2015	Sub‐cohort 5 2016–2018
Patients No. (%)	26,395 (100.0)	2737 (10.4)	5868 (22.2)	8342 (31.6)	6054 (22.9)	3394 (12.9)
Calendar year of the first diagnosis	1996–2018	1996–2000	2001–2005	2006–2010	2011–2015	2016–2018
Sex						
Male	11,912 (45.1%)	1372 (50.1%)	2751 (46.9%)	3701 (44.4%)	2635 (43.5%)	1453 (42.8%)
Female	14,483 (54.9%)	1365 (49.9%)	3117 (53.1%)	4641 (55.6%)	3419 (56.5%)	1941 (57.2%)
Age at the time of diagnosis						
Mean age, years (SD)	38.2 (13.0)	42.1 (12.2)	40.7 (12.6)	38.7 (13.0)	36.1 (13.0)	33.7 (12.1)
<20 years	1868 (7.1%)	135 (4.9%)	322 (5.5%)	563 (6.7%)	540 (8.9%)	308 (9.1%)
20–39 years	12,582 (47.7%)	971 (35.5%)	2364 (40.3%)	3849 (46.2%)	3252 (53.7%)	2146 (63.2%)
40–59 years	10,942 (41.5%)	1487 (54.3%)	2976 (50.7%)	3602 (43.2%)	2032 (33.6%)	845 (24.9%)
≥60 years	1003 (3.8%)	144 (5.2%)	206 (3.5%)	328 (3.9%)	230 (3.8%)	95 (2.8%)
First diagnosis from inpatient care, No. (%)	6920 (26.2)	1159 (57.0)	2180 (37.2)	1638 (19.6)	971 (16.0)	572 (16.9)
Patients included in the time points, No.						
3‐month time‐point	25,375	2608	5643	8111	5917	3096
1‐year time‐point	24,341	2573	5608	8083	5903	2174
2‐year time‐point	22,740	2507	5504	7965	5823	941
3‐year time‐point	21,569	2460	5432	7875	5778	24
4‐year time‐point	20,409	2419	5346	7787	4857	0
5‐year time‐point	19,078	2359	5257	7706	3756	0

The amount of people diagnosed with bipolar disorder was highest 31.6% (*N* = 8342) in the sub‐cohort 3 diagnosed in 2006–2010 and the lowest 10.4% (*N* = 2737) in the sub‐cohort 1 diagnosed in 1996–2000. The proportion of men diagnosed reduced by each sub‐cohort as time went by (50.1% in sub‐cohort 1 and 42.8% in sub‐cohort 5).

The mean age at the time of first recorded diagnosis also decreased in each sub‐cohort: among patients diagnosed in 1996–2000 (sub‐cohort 1) the mean age was 42.1 years (SD 12.2) whereas among patients diagnosed in 2016–2018 (sub‐cohort 5) it was 33.7 (SD 12.1).

### 3.2 General trends in medication use during the 5‐year follow‐up period

**FIGURE 1:**
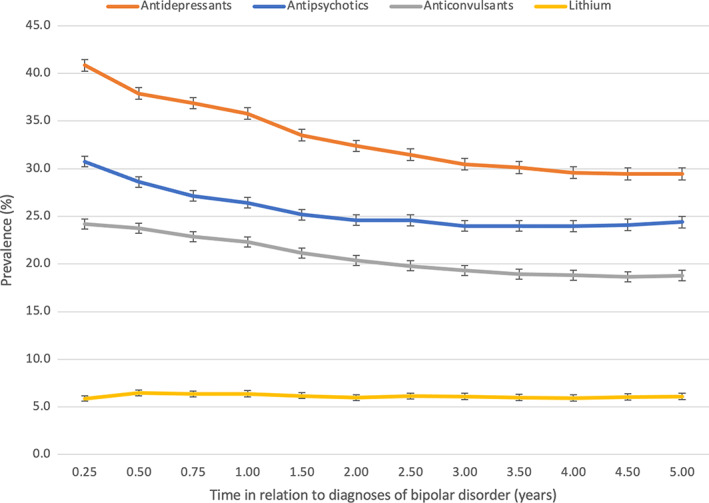
Prevalence of antipsychotic, antidepressant, anticonvulsant and lithium use from 3 months to 5 years after diagnoses of bipolar disorder. Note the change in x‐axis scale (quarter of the year during the first year and half of the year after the first year). The same person may have used more than one medication class.

The prevalence of overall medication use for the treatment of bipolar disorder declined steadily over the 5‐year observation period following the first recorded diagnosis (Figure 1). The three main medication classes used for the pharmacological treatment of bipolar disorder—antidepressants, antipsychotics, and mood stabilizers (divided into anticonvulsants and lithium)—all followed this similar trend of reducing prevalence of use. In general, of the medication classes, antidepressant use was significantly more prevalent during the follow‐up as compared to use of antipsychotics and mood stabilizers.

**FIGURE 2:**
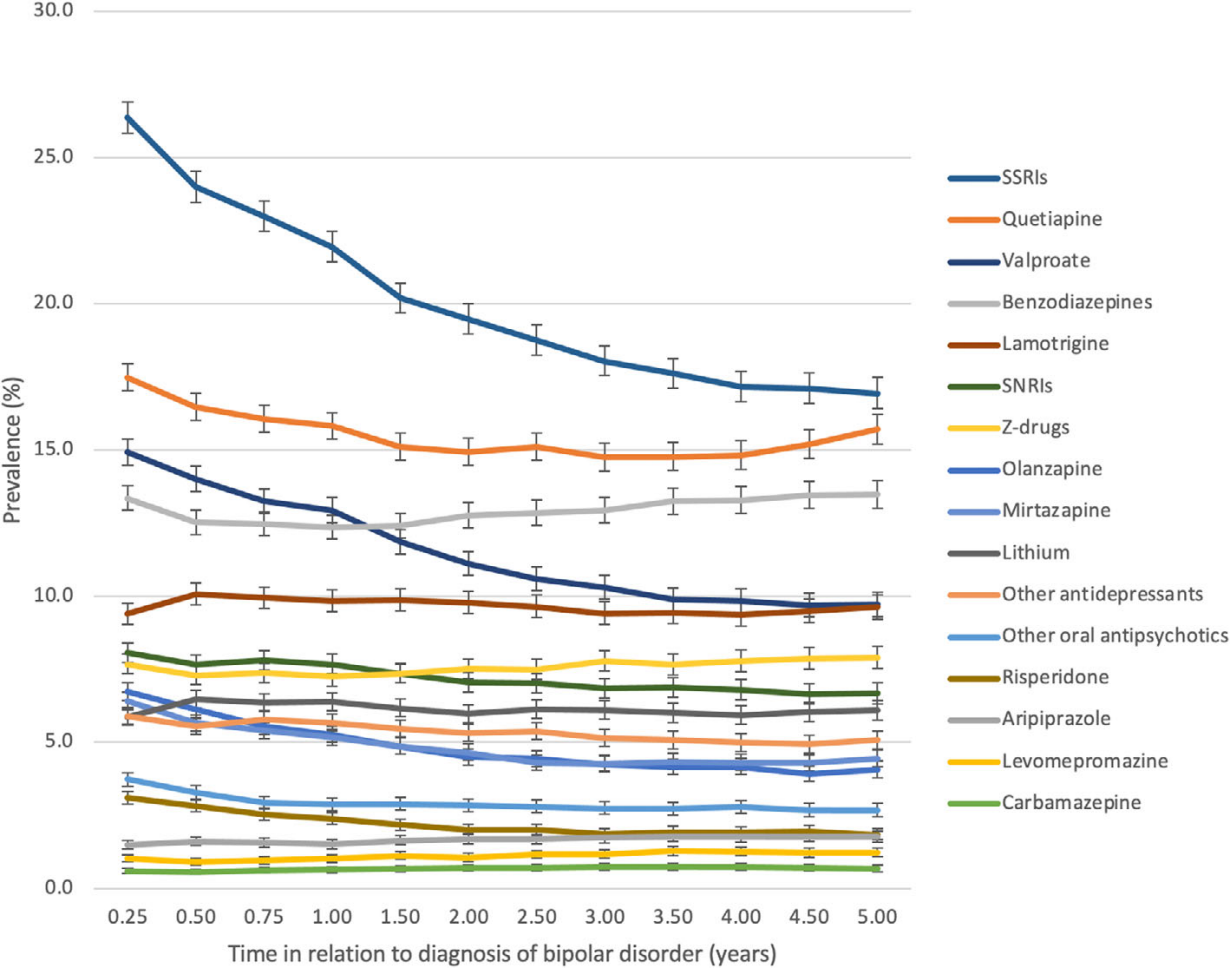
Prevalence of medication use from 3 months to 5 years after diagnoses of bipolar disorder. Note the change in the x‐axis scale quarter of the year during the first year and half of the year after first year). The same person may have used more than one medication class. SSRIs, selective serotonin reuptake inhibitors; SNRIs, serotonin‐norepinephrine reuptake inhibitors.

The prevalence of antidepressants use was 40.8% (95% CI 40.2–41.5) at the 3‐month time‐point and 29.5% (95% CI 28.8–30.1) at the 5‐year time‐point (Figure 1). During the 5 years following diagnosis, SSRIs were the most used antidepressant class, the prevalence being 26.4% at the highest (3‐month time‐point) and 16.9% at the lowest (5‐year time‐point) (Figure 2). Second most common antidepressant class used was SNRIs, the prevalence declining from 8.1% (3‐month time‐point) to 6.7% (5‐year time‐point) during the 5 years. The prevalences of mirtazapine and other antidepressants use were 6.4% for mirtazapine and 5.9% for other antidepressants at the 3‐month time‐point.

The prevalence of antipsychotic use was 30.8% (95% CI 30.2–31.3) at 3 months after diagnosis and 24.4% (95% CI 23.8–25.0) 5 years after diagnosis. The most common antipsychotic used was quetiapine, the prevalence declining from 17.5% to 15.7% during the five‐year follow‐up period. The second most common antipsychotic used was olanzapine, its prevalence being 6.7% at the 3‐month time‐point and 4.1% at the 5‐year time‐point (Figure 2).

The prevalence of anticonvulsant use declined from 24.2% (95% CI 23.6–24.7) to 18.8% (95% CI 18.2–19.3) during the 5‐year follow‐up period (Figure 1). The most commonly used anticonvulsant was valproate, the prevalence reducing from 14.9% (3‐month time‐point) to 9.7% (5‐year time‐point), followed by lamotrigine with relatively steady prevalence (ranging between 9.4% and 10.1%) as seen in Figure 2.

The prevalence of lithium use remained fairly steady (ranging between 5.9% and 6.5%) over the 5‐year follow‐up period (Figures 1 and 2).

The prevalence of benzodiazepine use reduced from 13.4% (95% CI 13.0–13.8) to 12.4% (95% CI 12.0–12.8) during the first year following diagnosis and then increased steadily to 13.5% (5‐year time‐point, 95% CI 13.0–14.0) during the rest of the follow‐up period. The prevalence of Z‐drugs followed a similar trend as benzodiazepines: during the first year the prevalence reduced from 7.7% (95% CI 7.4–8.0) to 7.3% (95% CI 6.9–7.6) and then increased to 7.9% (5‐year time‐point, 95% CI 7.5–8.3) during the rest of the observation period.

The cumulative use of the medication classes during the first year after diagnosis can be seen in Supplementary Figure 1. The cumulative first‐year incidence for antidepressants increased until the year 2007 after which it started to slope downwards. For antipsychotics, the cumulative incidence increased throughout the follow‐up period. For lithium, there was a larger initial decline in between 1996 and 2004 after which the first‐year cumulative incidence remained steady at around 10%. For other anticonvulsants there was an increasing trend until 2002, after which a decline until 2011.

### 3.3 Point prevalence of 3 months after diagnosis per sub‐cohort

**FIGURE 3:**
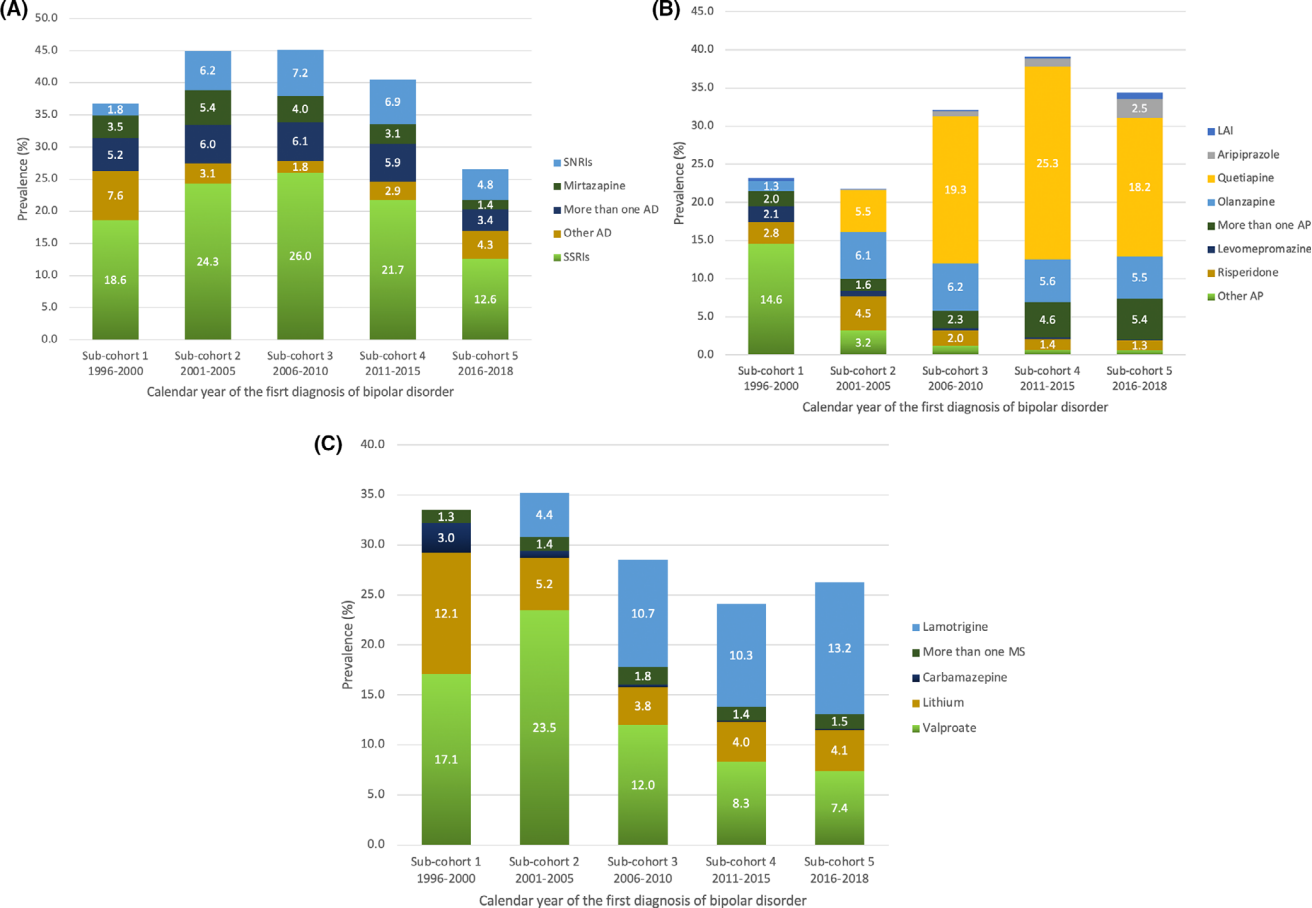
The prevalence of (A) antidepressant (AD), (B) antipsychotic (AP), and (C) mood stabilizer (MS) use 3 months after diagnoses in each sub‐cohort diagnosed in different years. SSRIs, selective serotonin reuptake inhibitors; SNRIs, serotonin‐norepinephrine reuptake inhibitors; LAI, Long‐acting injectable antipsychotics; More than one AD/AP/MS, use of two or more AD/AP/MS simultaneously.

The prevalence of antidepressant use 3 months after diagnoses stratified by calendar year of diagnoses are presented in Figure 3A. The highest prevalence was seen in patients diagnosed in 2006–2010 (sub‐cohort 3): 45.1% used antidepressants as part of their pharmacological treatment. The lowest prevalence of antidepressant use was among patients diagnosed in 2016–2018 (sub‐cohort 5, 26.5%). SSRIs were the most often used antidepressant class for all patients irrespective of the calendar year of diagnosis, the highest prevalence seen in patients diagnosed in 2006–2010 (sub‐cohort 3, 26.0%) and the lowest prevalence seen in patients diagnosed in 2016–2018 (sub‐cohort 5, 12.6%).

The prevalence of antipsychotic use among patients diagnosed in different calendar years are presented in Figure 3B. Antipsychotics were used the most among patients diagnosed in 2011–2015 (sub‐cohort 4), the prevalence being 39.1%, and the least among patients diagnosed in 2001–2005 (sub‐cohort 2), and the prevalence being 21.8%. In 2016–2018 (sub‐cohort 5) the prevalence of AP use was 34.4%. Quetiapine was the most used AP among patients diagnosed between the years 2006–2010 (sub‐cohort 3, 19.3%), 2011–2015 (sub‐cohort 4, 25.3%) and 2016–2018 (sub‐cohort 5, 18.2%). In patients diagnosed between the years 2001–2005 (sub‐cohort 2) olanzapine was the most common AP used (6.1%). Olanzapine remained to be the second most used AP in patients diagnosed after the year 2005 its prevalence of use being 6.2% at the highest (sub‐cohort 3).

The prevalence of LAI use was highest in patients diagnosed in 2016–2018 (sub‐cohort 5), although the prevalence was still only 0.8% (95% CI 0.6–1.2). The lowest prevalence of LAI use was seen in patients diagnosed in 2001–2005 (sub‐cohort 2, 0.1%) and 2006–2010 (sub‐cohort 3, 0.1%).

The prevalence of mood stabilizer use among patients diagnosed in different calendar years are presented in Figure 3C. Mood stabilizers were used the most among patients diagnosed in 2001–2005 (sub‐cohort 2), the prevalence being 35.2%, and the least among patients diagnosed in 2011–2015 (sub‐cohort 4), the prevalence being 24.1%. In 2016–2018 (sub‐cohort 5) the prevalence of MS use was 26.3%. In patients diagnosed in 1996–2000 (sub‐cohort 1), 2001–2005 (sub‐cohort 2) and 2006–2010 (sub‐cohort 3), valproate was the most commonly used MS, with the highest prevalence (23.5%) observed among patients diagnosed in 2001–2005 (sub‐cohort 2). The prevalence of valproate use reduced over the years and was only 7.4% in 2016–2018 (sub‐cohort 5). The use of lamotrigine increased over the years: lamotrigine was the most common MS used among patients diagnosed in 2006–2010 (sub‐cohort 3, 10.3%), and 2016–2018 (sub‐cohort 5, 13.2%).

Use of lithium early in the illness course (3 months after diagnosis) seemed to decline during the years, being used less and less in each sub‐cohort as time went by: the prevalence of lithium was the highest (12.1%, 95% CI 10.9–13.4) in patients diagnosed in 1996–2000 (sub‐cohort 1) and the lowest (4.0%, 95% CI 3.6–4.6) in patients diagnosed in 2011–2015 (sub‐cohort 4). In patients diagnosed in 2016–2018 (sub‐cohort 5) the prevalence of lithium was still only 4.1% (95% CI 3.4–4.8).

## 4 DISCUSSION

This large nationwide cohort study observed comprehensively time‐related trends in the real‐world pharmacological treatment of BD over the last two decades, during 5‐year observation periods after the first recorded diagnoses in Finnish patients. In our study the mean age at the time of diagnosis decreased substantially from 1996–2000 (sub‐cohort 1, 42.1 years) to 2016–2018 (sub‐cohort 5, 33.7 years) potentially reflecting a better recognition of the disorder. Studies have found that more than 70% of individuals with BD manifest clinical symptoms of the illness before the age of 25.^1^ However, it's common for BD patients to have on average 6–10‐year delay in diagnosis.^1^, ^3^

We identified several major issues regarding the clinical management of BD that can be summarized as follows: (i) the use of antidepressants was highly prevalent in the 5 years following diagnosis as well as in all the sub‐cohorts; (ii) use of lithium declined significantly among the sub‐cohorts diagnosed more recently and moreover, the prevalence of lithium use was remarkably low during the 5 years following diagnosis; (iii) LAIs were markedly underutilized; and (iv) benzodiazepines and Z‐drugs use remained significantly prevalent during the 5‐year observation period.

We observed the overall prevalence of antidepressant use to reduce from 40.8% (95% CI 40.2–41.5) to 29.5% (95% CI 28.8–30.1) during the 5 years following diagnosis. As the prevalences of antipsychotics and mood stabilizers were markedly lower, this suggests a proportion of patients were treated with antidepressants alone. Moreover, an alarming 45.1% prevalence of total antidepressant use at 3 months was seen in patients first diagnosed in 2006–2010 (sub‐cohort 3) and 45.0% in patients diagnosed in 2001–2005 (sub‐cohort 2). These findings are concerning and reflect an inadequate adherence to the evidence‐based treatment recommendations: as concluded earlier, the use of antidepressants alone is discouraged due to increased risk of treatment‐emergent mania, mood destabilization and suicide in patients with BD.^4^, ^6^, ^7^, ^10^ In addition, very limited evidence on the efficacy and safety of adjunctive treatment with antidepressants has been presented.^4^, ^10^ According to our data, the decline in the cumulative first‐year incidence of antidepressant use started in 2008, which coincided with the publication of the first national care guideline for bipolar disorder in Finland, which might have helped to steer medication practices towards more evidence‐based ones. However, the point‐prevalence of antidepressants was still 29.5% (95% CI 28.8–30.1) after 5 years of diagnosis despite that antidepressants are not recommended in the most recent CPGs or in research to be used for the long‐term treatment of BD.^4^, ^6^, ^7^, ^21^ Several other studies conducted in different countries, such as in Denmark, USA and United Kingdom, have observed similar trends in the frequency of antidepressant use^11^, ^12^, ^13^, ^18^, ^19^ indicating this issue not to be specific to clinical practice in Finland.

Reasons behind the overuse of antidepressants might include the natural course of the illness—patients with BD spend the majority of their non‐euthymic time in depressive episodes—and the lack of evidence‐based treatment options for bipolar depression as well as some inconsistency in recommendations regarding the prescription of antidepressants.^3^, ^8^, ^9^ Moreover, the relative ease of use and milder side‐effects might make newer antidepressants (such as SSRIs introduced during the 1990s) an attractive option for clinicians and patients as compared to mood stabilizers, antipsychotics and older antidepressants. Overall, the trend of replacing older medications with newer ones, seen for example with antidepressants and antipsychotics, likely reflects changes happening also on a general population level.

On the positive note, it is encouraging that in patients diagnosed in 2016–2018 (sub‐cohort 5) the prevalence of total antidepressant use had declined to 26.5% indicating an increased awareness to the evidence‐based treatment recommendations.

The use of lithium for BD patients significantly declined over the past two decades and lithium was also widely underused at least in the first 5 years following diagnosis. This finding is in line with several other studies^11^, ^12^, ^13^, ^19^ indicating the underuse of lithium to be an international issue in the clinical management of BD, to which several researchers have called an alarm.^22^, ^23^ Lithium is well studied and recognized for its efficacy in acute bipolar mania and maintenance treatment of BD due to its anti‐suicidal effects and prevention of mood episodes while improving long‐term course of illness^24^, ^25^ and is considered as the first‐line maintenance treatment for BD in most recent clinical practice guidelines.^6^, ^7^, ^14^

For these reasons, the findings of declining lithium use in new patients with BD and the under usage of lithium in the long‐term management of BD are not evidence‐based. Reasons that could potentially restrict the utilization of lithium are the possible adverse effects including kidney and thyroid function and the complexity of care compared to other medications. However, it is important to note that recent research shows the risk of developing chronic kidney disease in relation to lithium use to be significantly lower than previously thought.^26^, ^27^

We observed the prevalence of lithium use to remain steady during the 5 years following diagnosis, which might indicate that the patients who initially receive lithium treatment also stick to it for long‐term. This is an important notion, as it might indicate that concerns for side effects are over exaggerated as compared to clinical benefits. As per our study we can merely speculate on the reasons for the reducing use of lithium, and further studies are required to understand the underlying reasons behind the underutilization of lithium and to further encourage lithium use as the first‐line maintenance treatment unless contraindicated.

Interestingly, while lithium use declined, the use of other mood stabilizers—valproate and lamotrigine—and the antipsychotic quetiapine markedly increased, reflecting a replacement of lithium by these agents. These findings are in line with a previous population‐based and nationwide study on prescription patterns conducted in Denmark, which reported that lithium was prescribed less while the prescription of antiepileptic drugs and atypical antipsychotic drugs substantially increased.^19^

While all of these treatments are recommended by CPGs^6^, ^7^ there's evidence indicating lithium to be superior in efficacy in the long‐term.^28^, ^29^ However, lamotrigine is only suggested to be less effective in reducing the reoccurrence of mania compared to lithium and for the most part lamotrigine is considered as effective as lithium.^30^ This may support the increase of its prevalence of use.

Another notable observation was the underutilization of LAIs. In this study, we observed at the highest a 0.8% (95% CI 0.6–1.2) prevalence of LAI antipsychotic use. The efficacy of LAIs compared to that of oral antipsychotics (OAPs) is still understudied and relies on limited evidence.^31^, ^32^ However, the few studies available suggest LAIs (especially aripiprazole and risperidone) to be effective in delaying the time to relapse of any mood episodes as well as supporting adherence by ensuring consistent medication dose.^32^, ^33^, ^34^ In addition, LAIs were found to be significantly superior to OAPs in reducing the risk of rehospitalization in patients with BD.^34^, ^35^ This indicates that LAIs should be more frequently utilized in the management of BD instead of per oral antipsychotics, even though further research is warranted.

We observed the use of benzodiazepines and Z‐drugs to be markedly prevalent during the years following diagnosis. Importantly, 5 years after diagnosis the prevalence of benzodiazepine use was still 13.5% (95% CI 13.0–14.0) and that of Z‐drugs was 7.9% (95% CI 7.5–8.3) and these drugs were the only exceptions for the generally decreasing overall trend in prevalence of use. Evidence from research suggest the use of these drugs is associated with greater risk of reoccurrence of mood episodes^36^ and increased risk of suicide^37^ in patients with BD as well as impairment of cognition,^38^ all raising serious alarm. While the CANMAT and ISBD guideline considers the prescription of benzodiazepines to be appropriate in some cases when treating BD it emphasizes only short‐term utilization with a minimal dosage to be recommended and raises a caution regarding increased suicide risk, abuse and dependence.^6^ Our results indicate that their use is not limited to the short term and clinicians should make increased effort to try to rear their patients away from benzodiazepines quickly after initiation, or refrain from initiation.

On a final note, we would like to stress that although evidence‐based treatments should be used in general, the treatment of patients with bipolar disorder should always be individualized for each patient.

### 4.1 Strengths and limitations

Strengths of our study included a large nationwide sample of patients newly diagnosed with BD of all ages and with minimal exclusion criteria, including both primary care and specialized care, and a coverage of over 20 years of data on medication dispensings. The medication use was modeled with a well‐validated PRE2DUP method^20^ by using data from the Prescription register that cover pharmacy dispensings, correlating more straightforwardly to medication use in the real world than data based on prescriptions only. Therefore, our results reflect more reliably patterns of actual use of medications rather than clinicians' intentions to treat.

However, our study also has a number of limitations that need to be taken into consideration. Importantly, no patients were met or diagnosed by the study team, and therefore, all diagnoses are based on data collected from digital registers leading to a slight chance of misdiagnoses and selection bias of the study population that we were not able to control for. Moreover, there's an unavoidable possibility that some medications dispensed have not been used by the patients.

To conclude, our study leads to the following conclusions in light of clinical guidelines and recent research evidence on the effectiveness and safety of different medications for treatment of bipolar disorder: (i) the use of antidepressants is too prevalent, (ii) the use of lithium is declining and needs to be increased, and (iii) long‐acting injectable antipsychotics are markedly underutilized as compared to their oral counterparts.

## AUTHOR CONTRIBUTIONS

Concept and design: Juulia Poranen, Heidi Taipale, Jari Tiihonen, Markku Lähteenvuo. Acquisition, analysis, or interpretation of data: All authors. Drafting of the manuscript: Juulia Poranen. Critical revision of the manuscript for important intellectual content: All authors. Statistical analysis: Juulia Poranen, Heidi Taipale. Obtained funding: Jari Tiihonen.

## FUNDING INFORMATION

None.

## CONFLICT OF INTEREST

Jari Tiihonen, Heidi Taipale and Antti Tanskanen have participated in research projects funded by grants from Janssen‐Cilag and Eli Lilly to their employing institution. Heidi Taipale reports personal fees from Janssen‐Cilag and Otsuka. Jari Tiihonen reports personal fees from Eli Lilly, Evidera, Janssen‐Cilag, Lundbeck, Mediuutiset, Otsuka, Sidera, and Suvovion; and is a consultant to Orion and HLS Therapeutics. Markku Lähteenvuo is a board member of Genomi Solutions Ltd., Nursie Health Ltd., and Springflux Ltd. has received honoraria from Sunovion, Orion Pharma, Lundbeck, Otsuka Pharma, Recordati, Janssen and Janssen‐Cilag and research funding from the Finnish Cultural Foundation and the Emil Aaltonen Foundation.

### PEER REVIEW

The peer review history for this article is available at https://publons.com/publon/10.1111/acps.13504.

## Supporting information


**Supplementary Figure 1** Supporting Information.

## Data Availability

The data that support the findings of this study are available from the Finnish National Institute for Health and Welfare, the Social Insurance Institution of Finland, Finnish Centre for Pensions and Statistics Finland. Restrictions apply to the availability of these data, which were used under license for this study. Data are available from the authors with the permission from FinData, Finnish Social, and Health Data Permit Authority.
