# Supplementary figures and images for: Twenty‐year medication use trends in first‐episode bipolar disorder
Source: Acta Psychiatr Scand. 2022 Oct 12;146(6):583–93. doi: 10.1111/acps.13504 (PMC9828455; doi:10.1111/acps.13504)

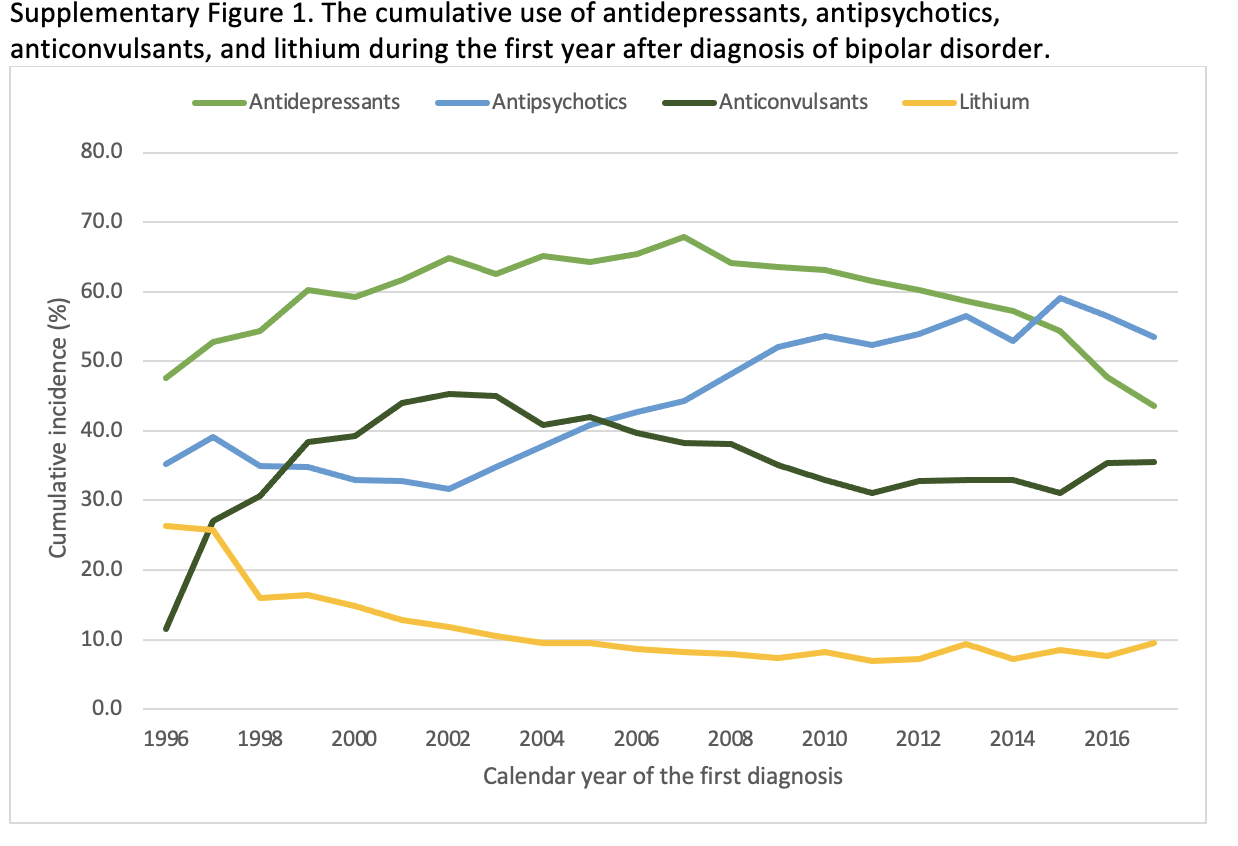

Supplement: Supplementary file 1 — Supplementary Figure 1 Supporting Information. [file ACPS-146-583-s001.png]
